# Open Access Accumulation Chambers SAGE (Surface-Air Gas Exchange)—DIY Philosophy

**DOI:** 10.3390/s26041384

**Published:** 2026-02-22

**Authors:** Bartosz Marian Zawilski, Vincent Busitllo

**Affiliations:** CESBIO, Université de Toulouse, CNES, CNRS, INRAE, IRD, 18 Avenue Edouard Belin, 31401 Toulouyse CEDEX 09, France; vincent.bustillo@utoulouse.fr

**Keywords:** closed-chamber, soil and water respiration, surface gas exchanges, autonomous automatic chambers

## Abstract

**Highlights:**

Soil and water gas exchanges with the atmosphere can be monitored using the closed-chamber technique. In this paper, we describe our open-access automatic closed chamber that can be operated on the soil and water surface with several options and configurations.

**What are the main findings?**

- Design of an open-access, versatile, automatic closed-chamber system for soil and water gas exchange measurements.
- Fully autonomous chamber description.

**What are the implications of the main findings?**

- Cost-effective deployment of dense closed-chamber networks.
- Improved accessibility of long-term greenhouse gas flux measurement.

**Abstract:**

Gas exchange between soil or water surfaces and the atmosphere is one of the main sources of greenhouse gas production and absorption. Faced with global climate change and increasing atmospheric concentrations of these gases, significant scientific efforts are being made to monitor this exchange using various techniques, including closed-chambers. Although relatively simple, this technique requires careful attention to several key points. Furthermore, any installation using commercial chambers is relatively expensive. Indeed, given the specific variability of gas exchange, a single chamber cannot assess all the gas exchange in the soil of a given plot. Several chambers are therefore necessary, which increases the overall cost of the installation. In our laboratory, we have built different types of chambers: portable “nomad” ultra-low-cost chambers for punctual, large-area measurement campaigns and “automatic” cost-effective chambers for long-term installations. In this article, we aim to share our experience by describing our achievements and providing a link to the complete documentation, which includes 3D and 2D plans, Gerber files for manufacturing printed circuit boards, and a parts list.

## 1. Introduction

Unfrozen soils emit nearly ten times more CO_2_ annually than global fossil fuel combustion. Due to warming soil temperatures and the consequent increase in microbial activity, this production is increasing by about 0.1% per year [1]. Other greenhouse gases (GHGs) such as nitrous oxide *N_2_O* (nearly 300 times the warming potential of carbon dioxide), or methane *CH_4_* (28 times the warming potential of carbon dioxide), are also produced mainly in the soil and in the aquatic environments [2,3]. Closed-chambers can also be used by the industry for geological prospection or gas leak detection [4,5]. In general, any gas exchange between the soil or water surface and the atmosphere can be monitored using the closed-chamber technique with an appropriate gas analyzer. There are several techniques used to monitor soil and water gas exchanges. Two main techniques are Eddy Covariance (EC) [6] and closed accumulation chambers [7]. Eddy Covariance is well-adapted for net flux monitoring, including soil, water, and vegetation contributions, whereas closed-chambers are well-adapted to measure the soil or water fluxes only. Eddy Covariance needs to have turbulent conditions to work well whereas closed-chambers do not. Consequently, during the night, Eddy Covariance is often inoperable as there is no wind; however, the chambers work well. There is also a possibility of using a transparent lid with the chambers that does not stop photosynthetic activity when the chamber is closed, allowing for the inclusion of vegetation contributions. However, as we will describe later in this paper, there are some issues with this setup. The EC technique is a low-intrusive, relatively expensive technique that requires several environmental conditions and a complex setup and corrections for quality measurement. The closed-chambers technique is rather intrusive but is the simplest and much less expensive. However, as chamber measurements are very local, several chambers are necessary to monitor the overall plot activity. The installation cost then rises proportionally. Our lab has successfully entered into the DIY philosophy, significantly lowering research costs and allowing us to work with the chosen sensors and protocols. We share our achievements with the scientific community as our chambers are already produced in our laboratory (30 pcs) for several ICOS and RZA sites, and successfully reproduced by colleagues from other laboratories and universities. The advantages and disadvantages of DIY will be discussed. The possibilities and options of open-platform SAGE chambers will be presented.

## 2. Closed Accumulation Chambers Technique Principles

### 2.1. Theory

**Figure 1:**
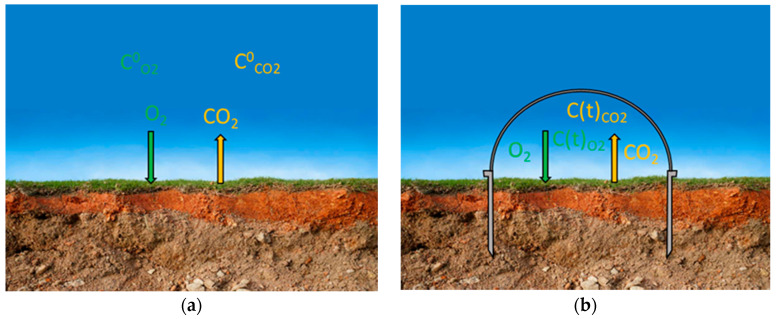
(**a**) Natural soil respiration. (**b**) Soil surface delimited by a collar, with the above volume closed by a chamber cloche (“bowl”).

The closed-chambers technique was described over one century ago [8] and relies on the gas accumulation or depletion over a well-delimited surface covered with a bowl. When some gases are exchanged between the concerned surface and the atmosphere (Figure 1a) with corresponding flux rates *F* (mol × m^−2^ × s^−1^), delimiting a portion of this surface *S* (m^2^) with a collar and hermetically closing a volume *V* (m^3^) above *S* (Figure 1b) will provoke gas concentration changes. Figure 1 schematizes the soil respiration process; it means soil oxygen uptake and soil carbon dioxide diffuse back to the atmosphere. If the carbon dioxide and oxygen fluxes, *F_CO_*_2_ and *F_O_*_2_, respectively, are assumed constant during the chamber closure, the closed-chamber internal concentrations follow a simple linear law.

(1) $$C_{CO2}\left(t\right)=C_{CO2}^{0}+t*F_{CO2}*S/V$$

for carbon dioxide and

(2) $$C_{O2}\left(t\right)=C_{O2}^{0}+t*F_{O2}*S/V$$

for oxygen, defining fluxes as positive when going from the soil or water surface to the atmosphere.

*C*^0^*_CO_*_2_ and *C*^0^*_O_*_2_ are the initial atmospheric carbon dioxide and oxygen concentration, and *t* is the chamber closure time. Of course, the reality is not so simple because the soil/water gases diffusion is not constant but depends on the surface upper-side atmosphere gas concentration. Then, in a closed chamber, as the gas concentrations change, the fluxes are not constant [9,10,11]. Consequently, gas concentrations in a closed chamber rather follow an asymptotic or exponential rise law:

(3) $$C\left(t\right)=C^{0}+\left(C^{L}-C^{0}\right)/(1-e^{-k*t})$$

*C_L_* is the equilibrium concentration (asymptotic concentration) almost reached when the chamber is closed for a long time, corresponding to the under-surface concentration, and *k* is a constant characteristic of the system. For homogeneity’s sake, if the concentrations are in mass/volume, the flux is in mass/surface. If the concentrations are in mol/volume, the flux is in mol/surface. Depending on the user’s preferences, instantaneous fluxes may be given in µmol/s/m^2^ or in µg/s/m^2^. The totalized fluxes may be given in kg/year/ha. The usual quantities reported by sensors and analyzers are not concentrations but mixing ratios in PPM or PPB. We need to transform it from mixing ratio to concentration using the perfect gas law, air temperature, and air pressure.

The flux is calculated by differentiating the concentration with respect to time at *t* = 0. For *CO_2_*, we obtain the following:

(4) $$F_{CO2}={\left(\frac{{dC}_{CO2}}{dt}\right)}_{t=0}*\frac{V}{S}=k\left(C^{L}-C^{0}\right)\frac{V}{S}$$

**Figure 2:**
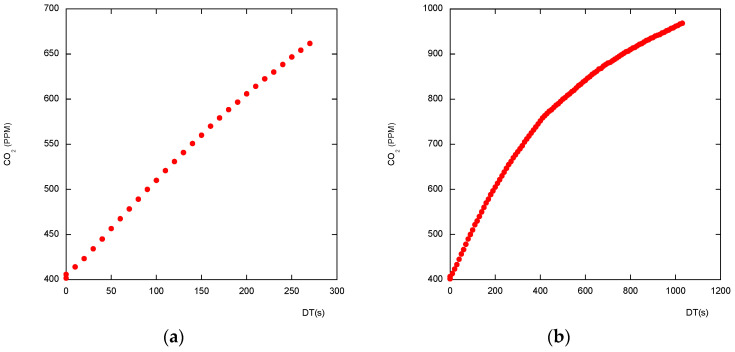
(**a**) Measuring during a short closure. (**b**) The same measurements were done during a longer closure. CO2 concentrations measured by an opaque cloche-equipped SAGE chamber using an external gas analyzer Li-840A on an agricultural tilled soil plot.

**Figure 3:**
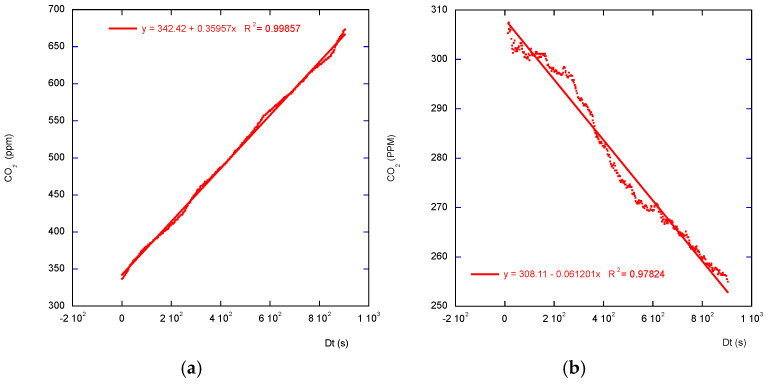
*CO_2_* concentrations measured by a transparent cloche-equipped SAGE chamber using an internal gas analyzer SCD-30 on a pasture plot (**a**) at night, (**b**) under the sun. A linear regression appears to be sufficient.

A detailed description of the flux derivative can be found on the Li-COR site [12,13]. For short measuring time and high effluxes, both laws are very close and appear linear (Figure 2). However, working with short measurement durations is unsafe, as at the beginning of the closure the ideal signal is mostly disturbed by the air-leading pipes’ gas transit time, gas homogenization by the internal fan time, sensors’ response time, and so on. Using linear regression and basing slope calculations only on the first measurements is subject to strong bias if the initial measurements are not discarded. Better results are given by longer chamber closure and asymptotic regression use. In the case of low gas permeability through the studied surface, and a resulting high studied gas concentration under this surface, such as in the non-tilled, high clay content soil, the linear shape of the studied gas increase may be long enough to discard the initial measurements and use a simple linear regression. This is probably the case of the studied pasture plot (Figure 3). Considerations about linear versus exponential regressions are well-described and documented in the literature [14,15].

Initial measured concentrations should be excluded, and the excluded duration depends on how fast the effective mixing is and how close the initial under-cloche air concentration is to the surface air concentration. Consequently, it depends on the under-cloche effective volume, the fan speed, and the initial under-cloche air flushing. Due to the possible gas stratification, when a chamber is flushed at a different height than the measurement height, the concentrations may be sensibly different. Li-COR chambers’ cloche movement is essentially horizontal, minimizing this problem. However, as SAGE chambers are made to work also on agricultural plots, surrounded by vegetation, horizontal movement is prohibited. We recently implemented a new optimized flushing protocol that consists of flushing the chamber when it is only 10% open. Thus, the chamber is flushed with the surface air. Water surface exchanges measurement imposes waterwings use and consequent collar dead volume increase. The resulting initial air mixing duration is longer, one minute in our case. The overall measurement duration is a compromise between the measurement shortness and the real fluxes. Indeed, as the chamber’s measurements are invasive, they should be as short as possible, but the data should be exploitable anyway, hence the compromise. There is no universal setting; it should be adapted to the real conditions. In practice, each raw dataset must be examined to identify the portion of the measurement where linear or exponential regression yields an optimal coefficient of determination R^2^. Automating this data post-processing is a complex task, and each scientist uses their own preferences.

Asymptotic regression is generally preferable for modeling the chamber measurements. However, when an almost-linear evolution is observed, a linear regression gives fewer errors on individual regression parameters.

For consequent efflux, even a longer closure on low-porosity soil may give almost-linear data. Figure 3a shows the real set of data collected by a chamber with an opaque cloche and SCD-30 sensor. A simple linear regression is of a rather good quality (R^2^ > 0.998).

### 2.2. Pros and Cons of the Closed-Chambers Technique

This technique is relatively simple and direct. The spatial delimitation is strong, which may be considered as a drawback but also as a benefit depending on what the measurement target is. However, its intrusive character is consequent. The collar presence perturbs soil life (root cutting) and may provoke serious gas leaks if used in a cracking vertisol. Chamber presence causes wind and rainfall perturbations, and soil gas exchanges are strongly correlated with external conditions. When the chamber is closed, the internal atmosphere is not in the same condition as the external atmosphere conditions. Mainly, the wind is stopped, and the internal air is homogenized by a fan or other mixing device [16].

These perturbations are not always well understood and cannot always be well quantified. We should be aware of it; there is no ideal measurement technique, and all approaches have advantages and limitations.

### 2.3. DIY Philosophy

Each profession has its own tools and apparatus. Scientists are no exception to the rule. We need our sensors and devices. Particularly in environmental science, as the measurement area is the whole Earth with its various climates, soil/water usage, and so on, we need a lot of measuring devices to acquire a global understanding. Our budget is not infinite; our needs are so various. The question to which we have to answer is the following: can we make our own device corresponding exactly to our own needs? And the answer we found was “Yes, we can”. The conceived scientific devices are made by scientists for scientists. In 2021, an official program called TERRA FORMA brought together interdisciplinary French scientific teams developing a set of low-tech open-source instruments to offer new environmental measurement capabilities using a multi-messenger approach (high spatial and temporal frequency). We are part of TERRA FORMA.

As always, there are pros and cons to building your own sensors. The pros are that, simply, what you are looking for does not exist on the market; if you are making your own device, nobody will impose their design and way to work with it. As is often the case when making your devices, the necessary budget drops compared to commercial devices. Also, there is no better way to understand how “it” works than make “it” yourself. Not only is it possible to build a system that fits exactly the needs but also to implement a chosen measurement protocol. If you do so, you become totally autonomous, and you do not have to rely on commercial offers to acquire, maintain, and troubleshoot your devices. The cons are the same or so; it is not sufficient to spend money to have a device, to maintain it, or to troubleshoot it. It needs some more work.

Concerning the closed-chambers, our laboratory uses two types of chambers. For a punctual campaign on large areas without any infrastructure, we are using the ultra-low-cost nomad chambers, which are described in our previous paper [17] and for which we are planning to add exploded mounting schemes. For long-installation chambers working automatically on an instrumented plot, we are using the chambers that are described in this paper. Designing these chambers requires important work. To save time for our colleagues, we are sharing all the plans (2D and 3D), parts list, PCB Gerber files, and pictures of the real chambers under Creative Commons license 4.0 (Attribution and Share alike) to allow other scientists to reproduce them URL (https://doi.org/10.5281/zenodo.18679255 (accessed on 13 February 2026)).

## 3. Automatic SAGE Chambers

These chambers are designed for long-term installation, working automatically, and do not require human presence. Numerous options and configurations are possible. While SAGE chambers can be used exactly as a usual commercial closed chamber, several configurations are not available in the commercial systems.

− Entirely autonomous chambers (sensors, datalogging, and power) for over one month of operation with a relatively small battery without a solar panel or indefinitely with an individual solar panel.
− Water surface gas exchange monitoring ability.
− Anti-pinch grids.
− Programmatically adjustable internal air mixing fan speed.

Also, SAGE chambers can be considered as a platform that accepts a multitude of sensors chosen by the users themselves.

### 3.1. Chambers’ Design

Closed-chamber design needs to respect several critical points. The geometrical shape needs to be cylindrical or, better, semi-spherical to allow good internal air homogenization without dead space, such as corners in a cubical shape [18]. Internal chamber air should be continuously homogenized when the chamber is closed. A proper sealing between the chamber cloche and the collar is necessary, and internal pressure has to be maintained equal to the external pressure [19,20,21,22].

### 3.2. Electronic and Mechanical Construction of SAGE Chambers

The target was to build a durable closed chamber compatible with ICOS requirements [23] that can be reproduced by anyone, without any specialized electronic knowledge or machining skills. Everyone who can weld a through-hole resistor or drill and tape a hole in an aluminum part can build that chamber. The second target was to use as many commercial parts as possible, such as a stainless-steel salad bowl for a chamber cloche, and sensors or electronic devices mounted in widely available commercial modules to avoid unnecessary work, cost increase, and to allow a very simple replacement process. The third goal was to make a chamber that can be used for a wide range of measurement needs, soil, water, opaque cloche, or a transparent one, with several options such as anti-vegetation pinch grids. Special care was taken for the chamber installation and maintenance and for troubleshooting ease.

#### 3.2.1. Electronics Main Board Modules

**Figure 4:**
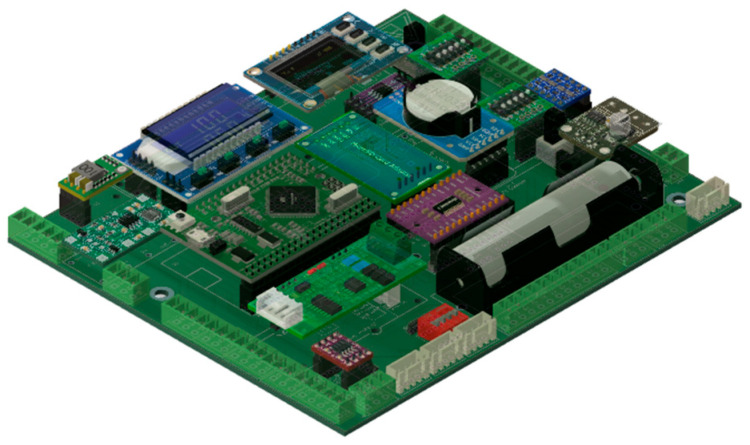
Main PCB version 03/25.

All electronics modules are widely available and require only a connector (female pin header or a strap) soldered on the main PCB (Figure 4) or under-cloche PCB. Besides the modules’ connectors, there are also wire connectors, a few diodes, resistors, and some exclusion network resistors used to form a simple voltage divider. All parts are through-hole mounting, not surface mounting, and do not require any special tools. PCB can be ordered online. It is only two layers, which makes it rather low-cost with a comfortable pin pitch to make soldering and wire mounting easy. The main used electronic modules are as follows:

- The used microcontroller is a Mega Pro Mini programmable using C and C++ under Arduino IDE, fully compatible with Arduino Mega 2560.
- To be able to use a 24 V power line, there are two dc-dc step-down modules. The first module, used only if a 24 V power line is actually used, delivers 12 V for the motor and, eventually, some sensors, such as the soil water content probe, through a solid-state relay (SSR) to be able to cut down the sensors’ power line and to save some energy when they are not used. This dc-dc is replaced by a jumper if the power used is a 12 V line. The second dc-dc step-down delivers 5 V to recharge an internal security battery and to power the microcontroller and all other modules, including the sensors, through another SSR.
- The air homogenization fan is pulse width modulation (PWM)-controlled, with an embedded tachometer (4-wire fan) to precisely control its speed of rotation, which is checked and recorded. For a transparent cloche, this fan is also transparent. PWM control is useful for changing fan rotation speed and adapting it to external wind conditions or other factors. This part of the measurement condition adaptations for *CO_2_* efflux is under test and will be described later. For evaporation measurement, because a closed chamber is also able to measure surface evaporation, the wind influence was already corrected and described [16].
- A PWM generator piloted by the microcontroller is implemented on the main board to achieve fan speed control.
- The embedded OLED display allows the showing of some info when required. The keys on the OLED module allow interaction with the microcontroller and, consequently, allow several pages and options to be displayed. This display should be cleared when not used to save some energy. The embedded microcontroller is able to manage it.
- Inside the chamber main box, there is an internal battery along with an uninterruptible power supply (UPS). When the main power is available, the battery is recharged. This battery serves to power the microcontroller and the SD card module in case of a main power shortage, allowing it to stop any actions while waiting for the power to be restored. It is a security device preventing SD card corruption held directly by an SD card module, relocated in an easy-to-access holder plugged with a ribbon cable to the SD module.
- Two LC filters to limit the dc-dc step-down module and motor noise.
- A Real-Time Clock module (DS3231-based) is used to hold the current date and time.
- Two delayed switches, along with a motor pilot (DRV8871 H Bridge-based), help to open or close the chamber when triggered by the microcontroller.
- A logic level shift is used between the microcontroller (5 V logic level) and the Luminox sensor (3.3 V logic level), even if it is not strictly necessary, as Luminox is 5 V logic-level-tolerant and the microcontroller “understands” 3.3 V logic-level UART inputs. This logic level shifter is also used to reserve one I^2^C line from the multiplexer (see further text) for a 3.3 V logic level.
- A communication module allows the use of RS-485 or another module, RS-422, for long-distance communication. There is also a reserved place and a connector for the LoRa WAN module. However, we kept in mind that LoRa allows long-range radio communication of low-density data, so the chambers’ raw data cannot be transmitted. However, the internally computed fluxes can be.
- An I^2^C line multiplexer is added to allow for multiple I^2^C devices to be used and to add several optional I^2^C devices without worrying about possible I^2^C address conflicts. Additionally, most I^2^C communication-based modules typically have their own pull-up resistors. Then, if there are too many modules on the same line, the resulting pull-up resistors are too weak. In other words, it is necessary to separate the I^2^C modules even if there is no address conflict.
- Because the under-cloche sensors are I^2^C and because this communication bus is not designed for long wires, a switch and connectors are placed on the main board to implement an optional I^2^C expander module based on the PB2B715 chip. This module allows the use of longer wires between the main board and the under-cloche board with sensors. However, with the high-quality wires we are using, our chambers do not require I^2^C expander use.
- As the optional GPS uses the SERIAL0 UART line of the microcontroller, when the GPS is used and the microcontroller is booting (may be booted by the watchdog), a blockage may occur. To solve this problem, a magnetic isolator based on ADUM1201 is inserted on the SERIAL0 line between the microcontroller and the GPS connector. This isolator is powered by the microcontroller, and when booting, the microcontroller is not powering it, setting the SERIAL0 line as disconnected. This prevents the boot-time blocking issue.
- Finally, a DIP switch block (4-pin) is used for address indication, allowing 16 hard-coded unique addresses. Indeed, each chamber has its own address.

#### 3.2.2. Mechanical Parts

**Figure 5:**
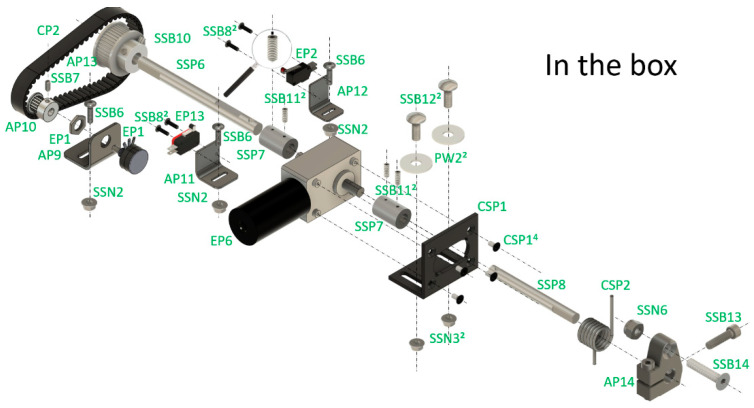
Example of the exploded mounting schemes.

Mechanical parts are made as often as possible from commercial parts. There is a need to drill these parts, sometimes tap them, paint them, and so on. Other parts are custom-made and can be ordered online, providing 2D and 3D drawings. We are including several exploded mounting schemes and descriptions to facilitate assembly, along with the 2D and 3D parts’ plans (Figure 5).

#### 3.2.3. Pricing

The price depends on where the parts are bought or made. For reference, a chamber made in France from electronic parts bought in an Asian online store and custom-made parts made in Maghreb (North Africa) is about 2000 €, which is about 1/3 of the commercial chambers’ price.

### 3.3. Options and Possible Configurations

SAGE automatic chambers are designed for various installation configurations and needs.

#### 3.3.1. Serial or Autonomous Configuration

**Figure 6:**
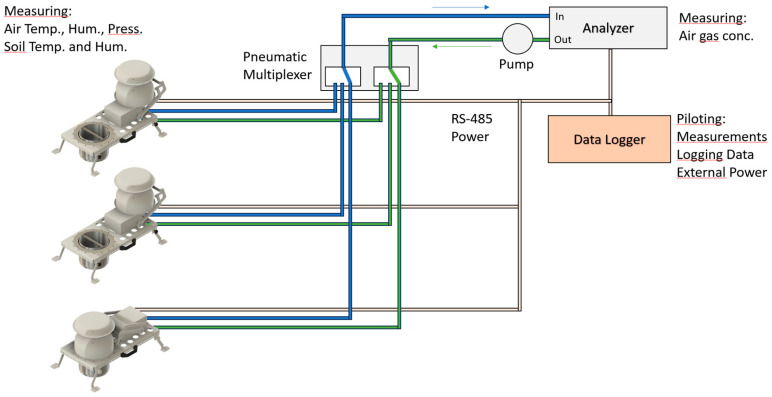
“Serial” configuration of the chambers with a unique air analyzer, a pneumatic multiplexer, and an external data logger, with data and power lines. Only one chamber can close at a time.

The same chamber can be used, as usual, in a network of several chambers connected to a pneumatic multiplexer and then to a single air analyzer (Figure 6), which is referred to as the “serial” configuration. This configuration is necessary for gas measurements such as nitrous oxide because an analyzer able to measure such small concentrations with such good precision is big and expensive. In other words, it is not realistic to equip each chamber with this kind of analyzer. Chambers are then connected to a pneumatic multiplexer, switching the pumped air sequentially from each chamber, injected into the analyzer, and driven back to the concerned chamber. This installation requires external piloting, which is usually done by a datalogger that pilots the multiplexer and chambers and reads data from the gas analyzer. SAGE automatic chambers have the possibility to attach two pipes for air pumping (IN and OUT) and use an RS-485 or RS-422 (both modules can be mounted on the main PCB) to communicate with an external datalogger using a simple protocol.

**Figure 7:**
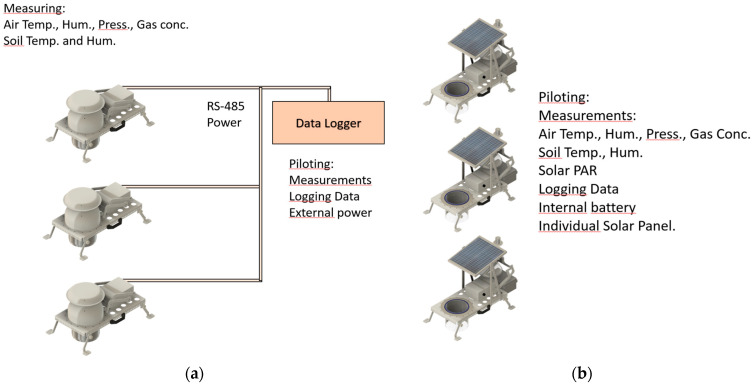
“Autonomous” configuration of the chambers, (**a**) with embedded sensors and an optional external datalogger, featuring a data log and power line. (**b**) All chambers are totally autonomous without data or power lines. In both cases, all chambers can close and measure simultaneously.

Another, much simpler configuration, so-called “autonomous” (Figure 7), used for the gases that do not require a unique analyzer lets us analyze gases directly under each chamber’s cloche without gas tubing, a pneumatic multiplexer, and a unique, expensive analyzer. Automatic SAGE chambers equipped with opaque cloche can use embedded sensors for *CO_2_* measurement (for example SCD30 from Sensirion), for *O_2_* measurement (for example Luminox from SST Sensing), for *CH_4_* measurement (TGS2611 from Figaro), and for *NH_3_* and H_2_S (SC05-NH3 and SC05-H2S from Shenzhen Shenchen Technology Co.). All these gases can be measured simultaneously; however, *H_2_S* presence can greatly affect *CH_4_* sensor readings. Additionally, each *CH_4_* sensor must be individually calibrated and corrected for air temperature and air moisture, which is a rather complex process described in the literature [24]. Of course, the embedded sensors can be replaced, and there are a lot of available small sensors that it would be impossible to list here; it is not our aim to suggest these sensors over others. We made our choice according to our experience; however, we did not check all of them. Air parameters such as air temperature, air moisture, and air pressure are sensed by the BME280 from Bosch. Internal air temperature and pressure are necessary to convert sensor-given measurements units, for example, Parts Per Million (PPM), to effective molar content (or grams). Air humidity sensing is useful to convert sensed gas quantities to dry mixing ratio, correcting the water evaporation effects. SAGE chambers equipped with transparent cloche can embed BME280 and *CO_2_*, *O_2_*, and *CH_4_* sensors only, as under a transparent cloche, the PCB should be small to avoid shadowing enclosed vegetation. However, for any special needs, everything can be adapted. Used *CO_2_* and *O_2_* sensors are the same as for our nomad chambers and are already described in our previous paper [17] and will not be reported here. The SCD-30, an NDIR sensor, was compared with a Li-7810 (Optical Feedback—Cavity-Enhanced Absorption Spectroscopy analyzer), showing its acceptable accuracy and stability. The Luminox-derived Apparent Respiration Quotient (ARQ) was checked against human respiration. The Luminox sensor is an optical sensor, not an electrochemical sensor, showing better long-term stability. *NH_3_* and H_2_*S* sensors were used as detectors rather than as quantitative sensors, as we are not able to check their calibration and long-term stability. These sensors are electrochemical and should be replaced relatively frequently. Please see the construction documentation. The *CH_4_* sensor can be used after individual calibration; however, because of the high cross-sensitivity with the sulfur compounds possibly present on our site, we finally discarded it. We can only recall that, obviously, the small, cheap sensors are not as accurate as a big, expensive analyzer. This reflects the expected trade-off between cost and analytical performance. However, on one hand, small sensors can rely on the same technology, such as Non-Dispersive Infrared (NDIR), showing similar long-term span stability. Their accuracy is often limited in terms of absolute value. For the closed-chambers, flux calculations are based on measured concentration variations, not on the absolute value. On the other hand, without fully autonomous chambers, sometimes even without a solar panel, the automatic chamber deployment is simply impossible. Among the cost-effective or low-cost sensors, some are acceptable while, obviously, others are not. Among the expensive sensors, some may still be unsuitable. At the same time, the notion “acceptable” is highly subjective. The closed-chamber measurement accuracy is directly affected by several factors:

− Soil or water flux stability. Any flux variation during the measurement biases the calculated main flux. This issue is very important for transparent cloche-equipped chambers as *CO_2_* absorption depends on the photosynthetic activity changing with the solar Photosynthetically Active Radiation (PAR). Any cloud passage or other shadowing is immediately perceptible in the recorded concentration.
− Gas concentration measurement uncertainty. This comes from the gas analyzers.
− Volume of the entrapped air during the measurements. This is effectively the adopted chamber system concern. Of course, the airtightness should be preserved. However, due to the pressure equilibration obligation, the internal volume can slightly change. Several commercial chambers use a capillary tube to expel surplus air. There is a tied Venturi effect problem [25] solved by Li-COR by a special venting design or by SAGE using a dilatation chamber made from a pneumatic silencer and a surgical nitrile glove that covers the output of the capillary tube. Water evaporation or changes in internal air temperature force a change in volume at constant pressure. If an external analyzer is used, this volume also includes the air-leading pipes, pneumatic multiplexer, and gas analyzer internal volumes. However, the main uncertainty comes from the soil surface. Indeed, the cloche is covering a collar inserted into the soil, and when the soil surface is not perfectly flat, it may be hard to estimate the volume inside the collar. The presence of litter on the soil surface induces porosity assessment, and vegetation presence imposes its volume assessment. Both assessments are rather approximate. This strong source of uncertainty concerns mostly the soil, not the water. It is a common closed-chambers issue.

Using air-leading pipes with closed-chambers may induce severe errors. Indeed, in case of any leak, errors can be important [26]. The leak occurs all the more, there are numerous pneumatic connections between chambers and multiplexer, between multiplexer and analyzer, or between analyzer and the pump.

Any possible leak in a connection is potentially disastrous, as the pressure is lower inside the pumped air tubes and higher inside the driven-back air tubes relatively to the atmospheric pressure. Inside the multiplexer, there is also a set of solenoid valves, which are a relatively fragile component that may leak and break down. The pump itself may also leak and break down. The installation with embedded sensors of the lowest accuracy may, all things considered, prove to be more accurate and more reliable. We have to consider several aspects: accuracy, reliability, power consumption, and installation complexity.

Another benefit of the embedded sensors is the possibility of simultaneous chamber measurement, allowing us to separate spatial variability from temporal variability. Indeed, with the unique analyzer installation, there is no alternative but to perform sequential measurements. In this case, the measurements coming from different chambers may vary because of chamber location (spatial variability) but also because of the chamber measurement period during the day (temporal variability). When the sensors are embedded under the cloche of each chamber, nothing prevents us from measuring them at the same time. Also, each chamber is equipped with 32 Gb internal memory, allowing us to log acquired data. If chamber closure is triggered every three hours for ten minutes, 32 Gb provides enough memory for 2000 years of measurements, which is rather comfortable. Data saved in the internal memory can be retrieved by RS-485 or RS-422, whichever is used, by direct USB connection or by physically removing the SD card, which is the fastest way. This SD card (micro-SD card, in fact) is accessible without chamber dismounting but is displaced into a waterproof SD card holder.

The embedded memory may serve to save acquired data but also to save a log file that includes any information helping to identify dysfunction, if any, and the configuration file.

It is a matter of internal soft programming, which is open to each user. In our laboratory, we chose to check and record results for each sensor at each microcontroller boot or wakeup. Any action required is logged and the result saved. If errors are detected, adapted checks are performed and results saved. This log file is very useful for troubleshooting.

#### 3.3.2. External Sensors

Auxiliary measurements, such as soil/water temperature or soil water content, are possible using several sensors. We are always adding soil/water temperature sensors based on the DS18B20 chip, soil water content when the chamber is used on the soil surface, and based on FDR technology with analog output. Other sensors can be added, including one analog and a few using I^2^C communication on six free I^2^C lines from the multiplexer (one of the lines is a 3.3 V logical level). Free I^2^C lines allow the addition of a few analog-to-digital converters (ADC), I^2^C to UART converters for serial communications, and so on, expanding evolution possibilities. An optional GPS (TTL communication) is foreseen, and its connector is optionally placed on the main box. The microcontroller manages it and records the chamber position when a measuring cycle is triggered. This possibility may be interesting when the chamber is freely floating on a lake. For additional sensors, a connector or cable gland should be added.

#### 3.3.3. Internal Data Logger and Battery-Powered Operations

As the chambers can be autonomous for gas analysis, the step to make them autonomous for functioning is relatively small. Everything is onboard, inside each chamber, to pilot its own actions according to the configuration file with the help of an embedded internal RTC. If a 7S3P Li-ion battery made with 4000 mAh capacity 18650 elements is attached, 6 weeks of the chamber’s functioning is possible (10-min cycle every 3 h) if the chamber enters deep sleep mode between each cycle. This mode is possible as the internal RTC has its own little rechargeable battery that can power it for years without recharging, and this RTC can wake up the microcontroller at a programmed date.

If an individual solar panel measuring 40 cm × 33 cm is added on the top of the chamber, the limitation of the chamber battery usage is greatly increased, but the limits are not yet known, as our setup was always working correctly during this year. Further testing under lower solar radiation and temperature conditions that may affect Li-ion battery performance would be required and lower temperatures. Whereas the used solar panel is sufficient for southern France, it may not be sufficient for Greenland.

Usual scheduling imposes a periodic cycle triggered every X hours. However, we can set SAGE chambers to trigger a cycle randomly with a minimum and maximum waiting time. In this case, if, in spite of this, we want to keep chamber closure all at the same time without any external data logger, we can set one chamber as the master, triggering the other chamber closure. But this is purely a software configuration issue, not a hardware problem, except for the need to connect all the chambers with a communication line.

**Figure 8:**
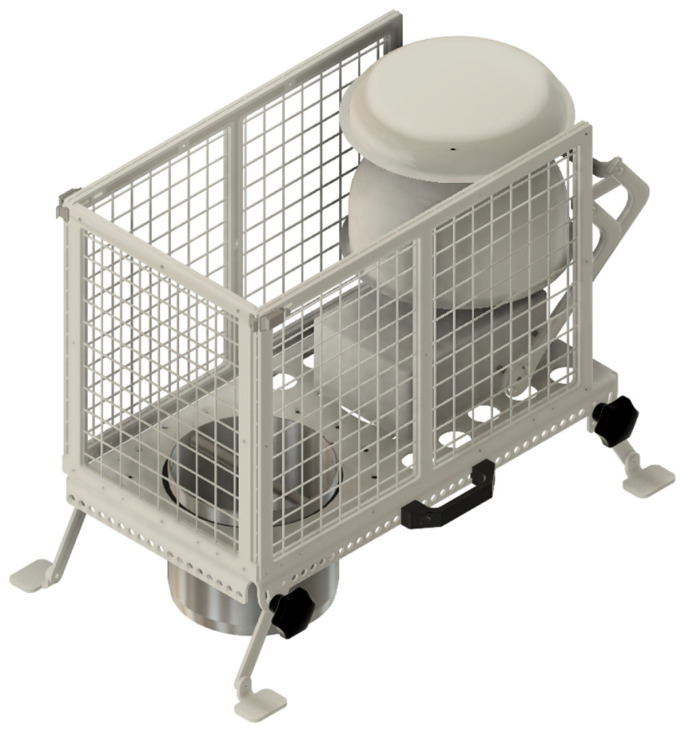
Chambers with anti-pinch grids.

For chamber installation on an agricultural plot sown, for example, with winter wheat or rapeseed, there is always a possibility that straw or other vegetation material gets pinched during chamber closure. In this case, measurements are not valid as the chamber airtightness is compromised. To prevent this situation, we can temporarily attach the anti-pinch grids (Figure 8).

#### 3.3.4. Opaque or Transparent Cloche

**Figure 9:**
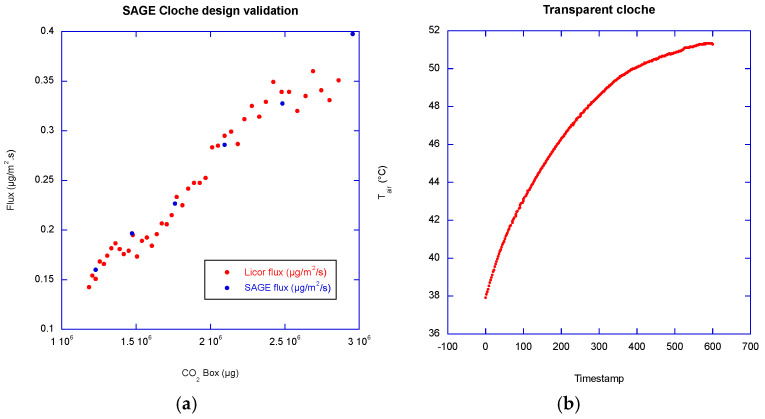
(**a**) Flux comparison given by the Li-810-104 chamber and the SAGE chamber. (**b**) An example of air temperature rise under a transparent cloche.

An opaque cloche is used when studying only soil or water, gas production/sink. To include photosynthetic activity, a transparent cloche is required. For this purpose, we use a PMMA cloche with a small printed circuit board holding sensors positioned beneath it, along with a transparent fan. A transparent cloche is never completely transparent, nor is the base of the chamber, and the collar, even if made of PMMA, is not entirely transparent. Condensation can also form inside the closed cloche [27], affecting the transmission of solar radiation. All these components block some of the solar radiation. As illustrated by Figure 9b, when using a transparent cloche, the temperature of the air trapped beneath it during the measurement cycle may rise rapidly, typically by 15 °C in 10 min under the summer-noon southern French sun. Therefore, it is important to monitor the air temperature and interrupt the measurement cycle if the temperature rise is deemed excessive. The temperature rise may compromise vegetation life and also the measurements made with internal sensors. Indeed, small sensors are usually temperature compensated; however, if the temperature is changing too quickly, the sensor compensation may not follow the air temperature. External sensors, such as Li-840A or the newest Li-850, heat up the analyzed air to a fixed temperature, making this setup much less sensitive to the air temperature changes. Furthermore, since photosynthetic activity depends on photosynthetically active radiation (PAR), which can vary during the measurement cycle due to cloud cover, it is necessary to add a PAR sensor and record the corresponding data. The chamber equipped with a transparent cloche must be oriented so that the printed under-cloche circuit board faces north; this orientation is necessary to prevent the under-cloche PCB and an eventual solar panel from casting a shadow on the measurement area.

When some vegetation is enclosed by the chamber’s collar, under the sun, during the chamber closure, the *CO_2_* concentration, resulting from respiration production and photosynthesis absorption, may decrease (Figure 3b).

#### 3.3.5. Soil or Water Monitoring

To our knowledge, no commercial chamber is specifically designed for use on the water surface. This is certainly a deficiency, as water also exchanges gases with the atmosphere, and until now, scientists have had to improvise to obtain the relevant measurements.

**Figure 10:**
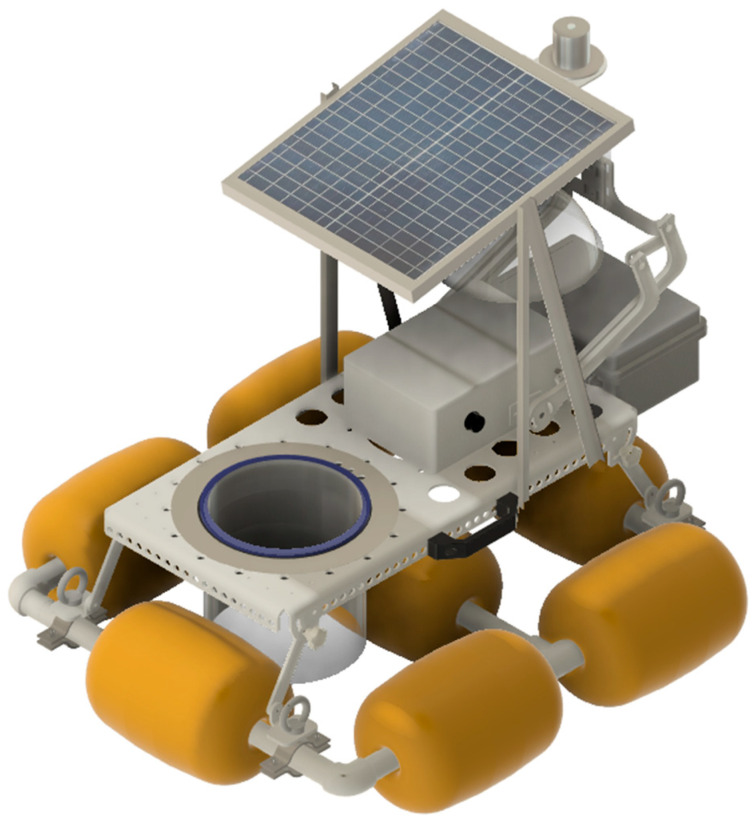
Floating chambers with a transparent cloche and collar, an individual solar panel, a PAR sensor, and a battery.

For monitoring water exchange with the atmosphere, waterwings made of standard PVC tubing and fittings, as well as fishing buoys, can be added (Figure 10). A standard chamber, usually equipped with a battery, is then attached to these waterwings. The stainless-steel collar is replaced with a PVC or PMMA collar. Figure 10 shows a SAGE chamber with a transparent cloche, equipped with a PAR sensor mounted on the solar panel and a battery housed in a waterproof casing at the rear of the chamber.

**Figure 11:**
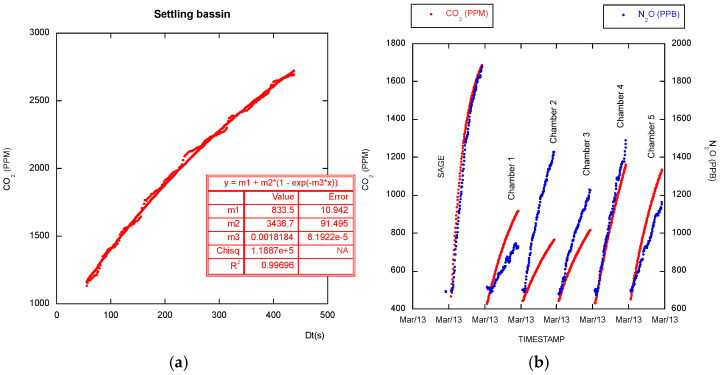
(**a**) Floating, opaque cloche-equipped SAGE chamber’s measurements on a settling basin using SCD-30 sensor. An asymptotic regression seems to be necessary for the water/atmosphere gas exchanges measures. (**b**) Six-chamber consecutive measurements in the serial configuration on an agricultural plot. The first chamber is SAGE; other chambers are aged, rectangular chambers. *CO_2_* is measured using Li-840A and N_2_O using Thermo Fisher 46i for all chambers in a serial configuration.

The described floating setup is suitable for relatively calm water surfaces but cannot be used on the ocean or rough waters. The *CO_2_* measurements shown in Figure 11a confirm that on high-permeability surfaces, which is the water surface case, an asymptotic regression should be used.

## 4. Discussion

It is challenging to report every possible configuration and all the available options, as existing options can be combined and new options can be added. It is an intrinsic advantage of a DIY achievement; the main limiting factor is our imagination. These open-access chambers may only benefit from being shared.

### 4.1. Reliability Check

SAGE chambers’ conception was refined by extensive testing, and Figure 3 and Figure 9 show a representative dataset illustrating operational reliability. The prototype was installed for over one year in real-world conditions and compared with another homemade chamber made by our colleagues from INRAe. Raw measurements differ because the volume-to-surface ratios of the SAGE and INRAe chambers are different. The SAGE time series appear smoother for both gases, likely due to improved air homogenization.

During a laboratory campaign, the SAGE chamber’s cloche design (Volume/Surface ratio ≥ 12.02 cm) was also checked against a Li-COR chamber, Li-8100-104 (Volume/Surface ratio ≥ 12.82 cm), at different fan speeds. Unsurprisingly, as shown in Figure 9, the measured fluxes were very similar at moderate fan speeds. Unfortunately, we were unable to use the Li-COR chamber on our agricultural plot as the plot soil has high clay content, and it was impossible to press in the plastic Li-COR collar. The SAGE collar is detachable and is made of stainless-steel for soil use, allowing hammering through a wood block. After these reliability checks, we added a thin stainless-steel plate on the base (noted SSP9 in the SAGE parts list) to improve the hardness of the contact surface between the base and the cloche, improving maintenance ease. We also replaced friction damping with viscous dampers (noted PP17), improving the damping robustness, and used a liquid thread lock on any rotating nut or bolt. Further tests consisted of checking the mechanical wear. Over one month, a short measurement cycle of one minute was triggered every 3 min. This corresponds to five-year functioning at eight cycles a day (every 3 h). We did not notice any premature wear.

### 4.2. Evolution

Battery-powered components imply maximal energy saving. If always turned on, sensors and the rest of the electronic components consume more power between the cycles than during the measurements. To be able to turn off every electronic device that does not need to be powered, we have had to modify the main PCB. Finally, to retrieve data from an autonomous chamber and its embedded data logger, it may take a long time. Then, to facilitate removal of the internal SD card, we displaced it into an easily accessible SD holder. The SD card can now be quickly changed without having to open the chamber’s main enclosure.

### 4.3. Actual SAGE Chambers

The described chambers can be used “as is” for various needs or can be completed and even modified. This is the advantage of “self-made” devices with open-access resources. SAGE chambers can be used with external sensors in a usual “serial” configuration without any modification. Internal sensors can be easily replaced with minimal modification of the under-cloche PCB. There is already an I^2^C, a UART line, and an analog-to-digital converter ADC for sensor interfacing. Analog sensors and I^2^C ones may also be used using the “spare” ports from the main PCB.

SAGE chambers have been deployed on an ICOS site and have operated continuously over an extended period, demonstrating their robustness and operating reliability. Currently, 12 other units are being deployed at RZA sites.

We are currently working to make SAGE chambers compatible with Li-COR systems, both older (analog) and newer (digital) generations. This would allow SAGE chambers to complement existing Li-COR installations, simply increasing the number of chambers that can be used. Moreover, this possibility exists, and Li-COR distributes the necessary documentation. To complete the serial configuration installation, we also will propose our open-access pneumatic multiplexers.

## 5. Conclusions

In this paper, we presented the SAGE automatic closed-chambers, an open-access, open-platform, and cost-effective system designed for long-term monitoring of soil and water surface–atmosphere gas exchanges. Rather than introducing new theoretical developments, this work focuses on practical design choices, reproducibility, and field-oriented implementation of an automatic chamber system compatible with ICOS requirements.

The proposed design combines mechanical simplicity, modular electronics, and a flexible software architecture, enabling both serial configurations using external gas analyzers and fully autonomous configurations based on integrated, low-cost sensors. Particular attention has been paid to the key methodological constraints of closed-chamber measurements, including air mixing, pressure balancing, chamber geometry, and operational robustness during prolonged field deployment.

By relying on widely available commercial components and by sharing all mechanical drawings, electronic schematics, firmware, and assembly documentation under a Creative Commons license, the SAGE chambers lower the barrier to deploying dense chamber networks. Beyond soil applications, the system also enables gas exchange measurements at the water–atmosphere interface, addressing a current limitation of most commercial chamber solutions.

Overall, the SAGE chambers illustrate how open-hardware approaches can complement existing commercial systems and contribute to more accessible, scalable, and transparent greenhouse gas monitoring infrastructures. Future work will focus on extended interoperability with reference commercial systems and on the continued development of the open-access system around SAGE chambers.

## Data Availability

The data are available under request.

## Abbreviations

The following abbreviations are used in this manuscript:

GHGs	Greenhouse gases
EC	Eddy Covariance
ICOS	Integrated Carbon Observation System
RZA	Réseau des zones atelier
SSR	Solid-state relay
PWM	Pulse width modulation
UPS	Uninterruptible power supply
SD	Secure digital
TTL	Transistor–transistor logic
UART	Universal asynchronous receiver transmitter
ADC	Analog to digital converter
PPM	Parts per million
NDIR	Non-dispersive infrared
ARQ	Apparent respiration quotient
PAR	Photosynthetically active radiation
